# Development of a mini pig model of peanut allergy

**DOI:** 10.3389/falgy.2024.1278801

**Published:** 2024-02-12

**Authors:** Akhilesh Kumar Shakya, Brittany Backus, Lazar D. Nesovic, Malini Mallick, Olivia Banister, Carla M. Davis, Sara Anvari, Harvinder Singh Gill

**Affiliations:** ^1^ Department of Chemical Engineering, Texas Tech University, Lubbock, TX, United States; ^2^ Department of Animal and Food Sciences, Texas Tech University, Lubbock, TX, United States; ^3^ Division of Immunology, Allergy and Retrovirology, Baylor College of Medicine, Texas Children’s Hospital, Houston, TX, United States

**Keywords:** allergen, allergy, anaphylaxis, animal model, food allergy, peanut allergy, porcine model, tape stripping

## Abstract

**Introduction:**

The prevalence of peanut allergies is increasing, emphasizing the need for an animal model to enhance our understanding of peanut allergy pathogenesis and to advance diagnostic tools and therapeutic interventions. While mice are frequently used as model organisms, their allergic responses do not fully mirror those observed in humans, warranting the exploration of a higher animal model. The porcine gastrointestinal system closely resembles that of humans, and exhibits allergy symptoms akin to human responses, making pigs a promising model for peanut allergy research.

**Methods:**

In this study we compared two allergen sensitization protocols involving either topical allergen application after repeated tape stripping (TS) or intraperitoneal (IP) injections to induce peanut-specific allergy and anaphylaxis reactions in mini pigs. Mini pigs sensitized with a combination of peanut protein extract (PE) and cholera toxin (CT) through either the IP or the TS route.

**Results:**

Sensitized pigs via both methods developed systemic PE-specific IgG and IgE responses. Following peanut challenge via the IP route, both TS- and IP-sensitized pigs displayed allergy symptoms, including lethargy, skin rashes, vomiting, and a drop in body temperature. However, respiratory distress was observed exclusively in pigs sensitized through the TS route and not in those sensitized through the IP route. However, it is noteworthy that both groups of sensitized pigs maintained peanut hypersensitivity for up to two months post-sensitization, albeit with a reduction in the severity of allergy symptoms. Importantly, both groups exhibited sustained levels of PE-specific IgG, IgE, and elevated concentrations of mast cell protease in their blood following the IP challenges.

**Discussion:**

Overall, this study reports TS and IP as two different modes of sensitization leading to onset of peanut specific allergic reactions in mini pigs, but only the TS-sensitization led to systemic anaphylaxis (simultaneous presence of symptoms: breathing difficulty, intense skin rash, and impaired mobility). A distinctive feature of these sensitization protocols is the 100% success rate (*N* = 4 pigs per group) in sensitizing the subjects.

## 1 Introduction

Peanut allergy is one of the most common types of food allergies. It affects millions of people worldwide and is the leading cause of anaphylaxis (1, 2). It is estimated that approximately 1% of the US population, especially children under five years of age, are living with peanut allergy (3). Strict avoidance of peanuts, patient education, and medications such as epinephrine that provide temporary relief are the mainstay of a peanut allergy management plan. There continues to remain a need to develop new and improved peanut allergy treatments, to better understand disease etiology, and to establish superior biomarkers.

The mouse model has been extensively explored in food allergy studies because of the ease of handling and availability of reagents (4). However, mice do not develop many human-like food allergy symptoms because their gut physiology and anatomy are different from humans. A model that mimics humans more closely would be a better choice. In this sense, pigs are noteworthy. The pig intestinal physiology is anatomically and histologically similar to humans (5) and the pig is also a good model to study intestinal immunology (6). Their microflora is more diverse than rodent models, and they are outbred like humans. These characteristics make pigs an important model because they can better recapitulate the human genetic variability as opposed to inbred mice. The pigs also resemble humans in terms of acute symptoms including diarrhea, vomiting, weight loss, cutaneous erythema, and respiratory distress (7, 8). Thus, the pig has been recognized as an animal model for peanut allergy (8, 9) and egg allergy (10).

However, there are limited studies exploring the use of pigs as a peanut allergy model, and in fact, we are aware of just two such studies—Helm et al. (2002) (8) and Rupa et al. (2008) (9). Helm et al. were perhaps the first to report a peanut allergy sensitization protocol in neonatal farm pigs. They injected crude peanut extract mixed with cholera toxin (CT) via intraperitoneal (IP) injection to neonatal pigs on days 9, 10, 11, 18 and 25 after birth. Upon oral challenge with peanuts, 8 of the 14 sensitized pigs (57%) were reported to present with grade 2 clinical symptoms including vomiting, lethargy, skin rashes, tremors, and convulsions; and 3 of 14 pigs were reported to require an epinephrine injection to manage the allergic reaction. Sensitized pigs were also reported to have developed a positive reaction following immediate hypersensitivity skin testing (8). Rupa et al. sensitized the pigs just thrice, once each on days 14, 21 and 35 after birth. They reported low to mild clinical symptoms after oral challenge, with 1 out of 5 animals requiring an epinephrine injection (9). Because of the low success rate of these protocols in generating peanut sensitized pigs and the high variability in the spectrum of allergy symptoms exhibited by them, their utility for translational studies is rather limited.

To address this limitation, here we report a method of generating a pig model of peanut allergy using two different sensitization protocols, one based on topical allergen application after repeated skin tape stripping (TS), and another based on IP injections. The unique feature is that in both approaches we achieved a 100% success rate in developing peanut sensitized pigs, and in eliciting peanut-specific allergic and anaphylaxis reactions. We believe this is also the first study to demonstrate that the TS approach can be used to induce peanut sensitization in a mini pig model.

## 2 Materials and methods

### 2.1 Animals and housing

Twelve, 3–4-day-old mini pigs (Yucatan Miniature Swine) were acquired from Sinclair Research BioResources (MO, USA) and acclimated for sixteen days before starting the study when pigs were 19–20 days old. Animals were housed in climate-controlled environment conditions. Pigs were provided *ad libitum* water. Mini pigs were fed on Birthright™ standard baby pig milk replacer (Product No.-8229, Ralco Agriculture Marshall, MN) until weaned from milk at approximately 18 days of age. Creep feeding started from 7 days old until 7 days post-weaning with a mini-pig starter diet (Lab Diets 5,080, Lab Diet, St. Louis, MO) and later switched to a mini-pig standard grower diet (Lab Diet 5L80, Lab Diet, St. Louis, MO). All diets were peanut-free, and animals were meal-fed to meet nutritional requirements. All animal experiments were performed under approved protocols and ethical guidelines of the Institutional Animal Care and Use Committee at Texas Tech University, USA.

### 2.2 Sensitization protocols

**Figure 1:**
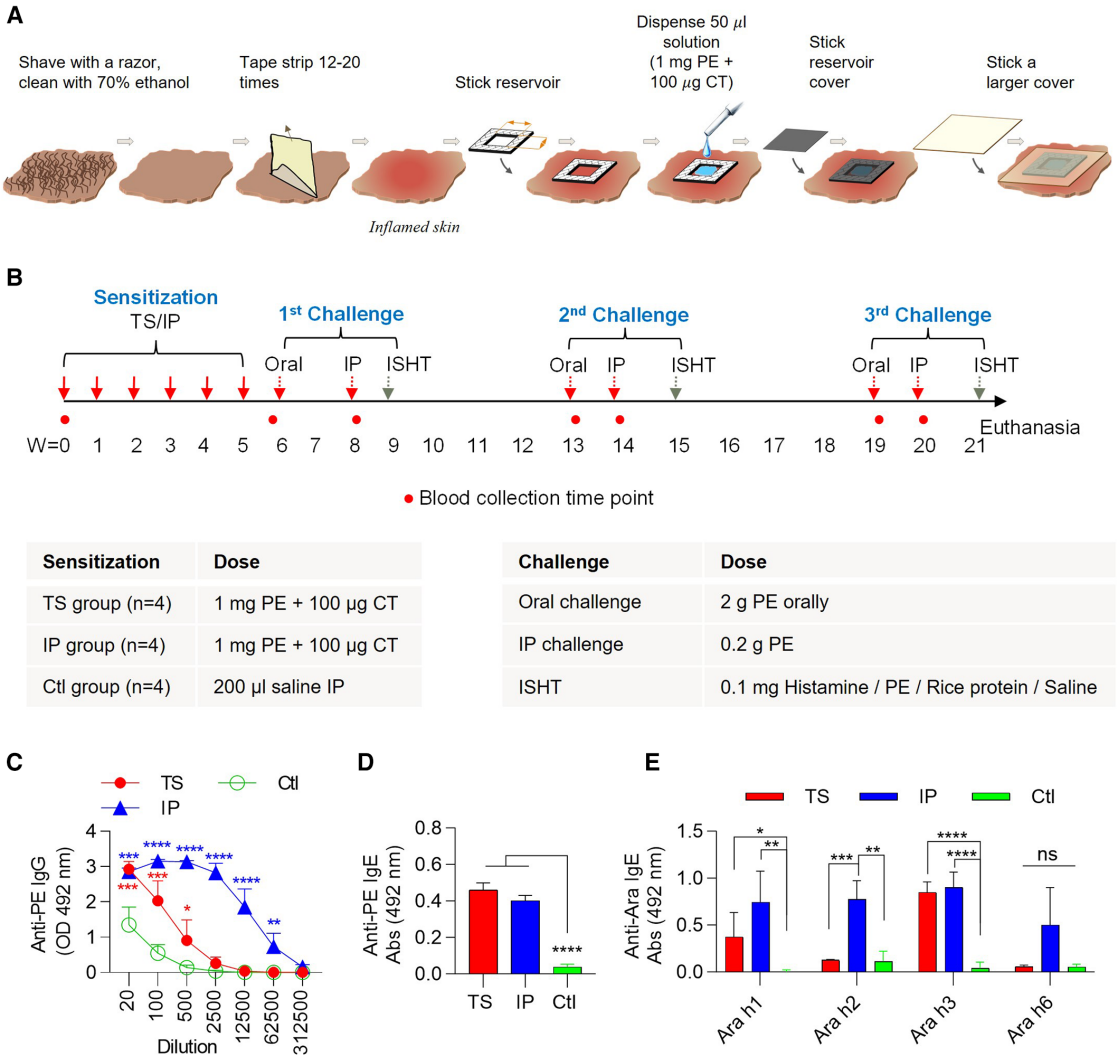
Sensitization protocol, experimental timeline, and plasma analysis after sensitization but prior to performing any challenges. (**A**) Illustration of skin TS procedure and peanut allergen application. (**B**) Schematic of experimental timeline. Mini pigs were sensitized with PE mixed with CT through IP injections or topical application on TS skin on a weekly basis for six weeks. One week after the last sensitization, all pigs were challenged (1st Challenge), which comprised first an oral challenge, next an IP challenge, and finally an immediate skin hypersensitivity test (ISHT). This “Challenge combo” was repeated two more times at about a one-month gap. At the end of 3rd challenge, pigs were euthanized. (**C–E**) Plasma analysis after sensitization but just before first oral challenge. (**C**) Anti-PE IgG at different plasma dilutions. (**D**) Anti-PE IgE antibody response at 1:20 plasma dilution. (**E**) Anti-Ara IgE at 1:20 plasma dilution. Individual pig plasma was used in analysis. The *t*-test was used to compare sensitized and control groups. Bars denote Mean ± SD. *p ≤ 0.05, **p ≤ 0.01, ***p ≤ 0.001,****p ≤ 0.0001, and ns: not significant. *N* = 4 pigs per group.

Pigs were randomly assigned to the TS group (female = 2, male = 2), IP group (female = 2, male = 2), or control (Ctl) group (female = 4). For IP sensitization, 1 mg peanut extract (PE) (Greer Laboratories, Inc.) mixed with 100 µg CT (Sigma Aldrich, MO) was injected every week via the IP route for six weeks in total. For TS sensitization, the sensitization procedure is summarized in Figure 1A. Briefly, the pigs were anesthetized with an isoflurane air mixture and subsequently a small portion of hair from their back was removed with the help of a shaving razor. Skin was then vigorously cleaned with 70% ethanol to remove dirt for proper adhesion of the tape. Subsequently, a tape (Comply™ Steam Indicator Tape, 3M) was stuck to the hairless skin and removed. This was repeated up to 12–20 times on the same skin area (measuring about 1 × 1 cm^2^) until a characteristic shine was observed on the skin. Tape-stripping is done to sequentially remove layers of the skin stratum corneum layer. This increases skin permeability and causes local inflammation. Next a reservoir was created using a tape (Acrysure Next Gen PE Foam, Mactac) measuring 1/16 inches in thickness. The outer dimensions of the reservoir were 1.2 × 1.2 cm with a central cavity measuring 1 × 1 cm. The reservoir was applied over the tape stripped skin with its inner cavity aligned and placed directly over the tape stripped area. A 50 µl liquid mixture of PE (1 mg) and CT (100 µg) was dispensed into the inner cavity. The inner cavity was then covered with a medical grade adhesive (Acrysure Next Gen Medical Grade Adhesive, Mactac) to confine the liquid in the cavity. Another larger tape (Waterproof Transparent Dressing, CVS or Tegaderm, 3M) was applied on top to better secure the reservoir to the skin. After this procedure, pigs were removed from anesthesia and monitored until awake. Pigs were kept on a heated bed throughout the entire procedure. The adhesive system was allowed to stay on skin around 24 h, and then it was removed. This procedure was performed every week for a total of six weeks.

### 2.3 Allergen challenge and assessment of clinical reactions

Oral challenge was performed with 2 g PE mixed in a frozen banana paste (made in house). One week after oral challenge, pigs were challenged through the IP route by injecting 0.2 g PE dissolved in 3 ml sterile saline solution. After each challenge, body temperature was recorded every 15 min for 2 h using a temperature sensor (LifeChip with Bio-Thermo Technology) implanted on the left side of neck muscle, and read with the help of a Universal Worldscan Reader (HomeAgain, Merck & Co., Inc.). Pigs were monitored for any clinical signs of allergic reaction and scored as discussed previously (8, 11). In brief, 0 = no symptoms; 1 = immobility, lethargy; 2 = scratching, rash, coughing, gagging, stomach contractions; 3 = diarrhea, emesis; 4 = increase in respiratory rate, neck extension; 5 = forced expiration; 6 = confluent cutaneous reddening, cyanosis, anaphylaxis. In this study, we categorized “systemic anaphylaxis’ by the simultaneous presence of three symptoms: difficulty in breathing, intense skin rash, and impaired mobility. Epinephrine (1 mg/ml) at 0.02 mg/kg, dexamethasone (2 mg/ml) at 0.22 mg/kg, and diphenhydramine (50 mg/ml) at 2 mg/kg body weight were given intramuscularly to manage severity of allergic reactions. Blood samples were collected before and after the challenge to measure molecular markers.

### 2.4 Blood collection and analysis

Whole blood was collected via jugular vein puncture into EDTA blood collection tubes, kept on ice until it was centrifuged at 10,000 × g for 15 min for plasma separation, and plasma was stored at −80°C for analysis. The ELISA technique was used to measure PE-specific antibodies in plasma. ELISA plates were coated overnight with PE at 50 μg/ml in 0.1 M carbonate buffer (50 µl/well) for PE-specific IgG and IgE detection. Separate plates were coated with natural Ara h1, h2, h3 and h6 (InBio, Inc., VA) at 10 μg/ml to detect Ara h specific IgE response in pig plasma. A 2% milk solution was used for the blocking step. To detect pig IgG, a goat anti-pig IgG HRP conjugate (BIO-RAD-AHP865P) was used. To detect pig IgE, a two-stage antibody system was used, comprising an unconjugated mouse anti-pig IgE (Cloud Clone Corp.-MAA545Po21) followed by an HRP-conjugated goat anti-mouse IgG (Southern Biotech-1030-05). O-phenylenediamine dihydrochloride in phosphate citrate buffer (pH 5.0) with H_2_O_2_ was used to develop color, which was read at 492 nm. Mast cell tryptase quantification in plasma was conducted following the guidelines provided by a commercially available ELISA kit (MyBioSource, Catalog Number MBS264821).

### 2.5 Immediate skin hypersensitivity test (ISHT)

ISHT was performed one week after each IP challenge and characterized as discussed previously (8). Briefly, animals were anesthetized, their flank region was first shaved using an electric hair trimmer and then with a razor blade. Four squares, each around 10 × 10 cm^2^ were drawn with a pen on the shaved flank. In each square, a tuberculin syringe was used to intradermally inject 100 µl of 100 µg PE, 100 µg histamine (positive control), 100 µg rice protein (irrelevant protein control), or saline solution (negative control). Fifteen minutes later wheal-flare inflammatory diameter (WFD) was measured with calipers and digital pictures were taken to observe the change in skin morphology.

### 2.6 Statistical analyses

Statistical analysis was conducted using Graphpad Prism 6 software. The *t*-test was used to compare the experimental groups. Significance was considered for *p* < 0.05 for a 95% confidence interval.

## 3 Results

### 3.1 Induction of anti-PE IgE antibodies post-sensitization

Pigs were sensitized through TS or IP routes for six weeks, and the plasma was analyzed one week after the last sensitization dose just prior to performing peanut challenge (Figure 1B). PE-specific IgG response increased significantly in both the TS and IP sensitized groups compared to the control group (Figure 1C). Notably, within the IP group, the IgG response surpassed that of the TS group. The PE-specific IgE response was also higher in both TS and IP sensitized groups, as compared to the control group (Figure 1D). Examination of IgE antibodies against the common peanut allergens, Ara h1, h2, h3, and h6 revealed that the TS route primarily stimulated IgE against Ara h1 and Ara h3, while the IP route stimulated against Ara h1, Ara h2, Ara h3, and weakly against Ara h6 (Figure 1E).

### 3.2 Clinical symptoms of allergic response to the oral and IP challenges

#### 3.2.1 Oral challenges

**Figure 2:**
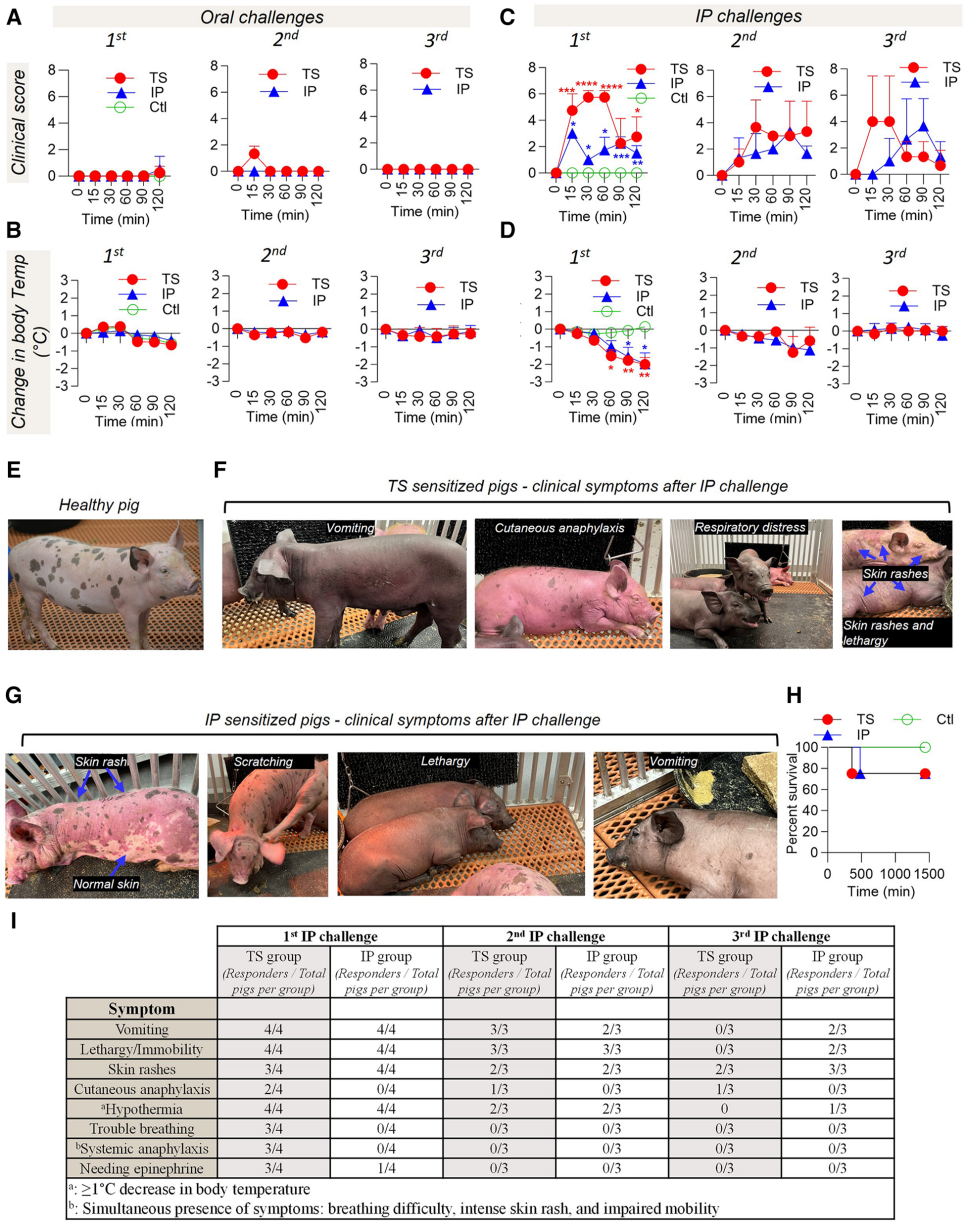
Allergic reactions after different oral and IP challenges. Sensitized pigs were challenged through the oral and IP routes at three different times. (**A**) Clinical score after oral challenges, (**B**) change in body temperature after oral challenges, (**C**) clinical score after IP challenges, and (**D**) change in body temperature after IP challenges. Digital pictures of different clinical symptoms observed in (**E**) a healthy pig, (**F**) TS-sensitized pigs challenged via IP route, and **(G)** IP-sensitized pigs challenged via IP route. (**H**) Percent survival after 1st challenge via the IP route. No loss was observed at 2nd and 3rd challenges. (**I**) Summary comparison of symptoms in TS and IP sensitized pigs after IP challenge. The *t*-test was used to compare sensitized and control pigs. Bars denote Mean ± SD. *p ≤ 0.05, **p ≤ 0.01, ***p ≤ 0.001 and ****p ≤ 0.0001. *N* = 4 pigs per group for 1st challenge. *N* = 3 pigs per group for 2nd and 3rd challenges. The control (Ctl) group is only presented in the 1st challenge. This is because, during the 1st challenge, the Ctl group experienced an oral challenge, IP challenge, and ISHT. Consequently, it is no longer deemed naïve to PE exposure and is no longer suitable to function as a Ctl group.

The TS and IP sensitized pigs were orally challenged with PE at three different time points about 6–7 weeks apart. No significant allergic reactions (Figure 2A) or notable changes in body temperature (Figure 2B) were observed following any of the oral challenges.

#### 3.2.2 First IP challenge

After each oral challenge, the pigs were challenged with PE via the IP route. Both TS and IP sensitized pigs responded and displayed clinical symptoms including anaphylaxis and a significant drop in body temperature. The clinical reactions observed during the first IP challenge were intense and severe. The clinical symptoms are discussed below in detail.

*Ctl group:* All control pigs were normal and healthy after the first IP challenge (Figures 2C–E) and presented neither of the allergic symptoms.

*TS sensitized group*: Severe allergic symptoms, including systemic anaphylaxis, were observed in the TS pigs during the first IP challenge. Five minutes post-challenge, all pigs started to vomit and presented symptoms of uneasiness and lethargy. Multiple episodes of vomiting were observed during the observation period of 120 min post IP challenge (Figures 2C,D,F). Thirty minutes after IP challenge, 50% of the pigs (two of four) displayed cutaneous anaphylaxis as their skin started to turn purple (Figure 2F), and 75% (three of four) demonstrated respiratory difficulty (Figure 2F) and a significant drop in body temperature (Figure 2D). Pigs displaying respiratory distress were immediately administered epinephrine and dexamethasone. Despite the interventions, one of the pigs (female) died due to severe hypothermia (Figure 2H). About 120 min after the IP challenge, the skin color of the pigs that showed findings of cutaneous anaphylaxis returned to a normal baseline color, however, they continued to suffer from drop in body temperature. After approximately 6 h following the IP challenge, the pigs regained their baseline body temperature (∼103°F) but remained lethargic with mild skin rashes. The next day, pigs were active and looked healthy without any allergic symptoms.

*IP sensitized group:* IP-sensitized pigs also showed strong allergy symptoms during the first IP challenge. All pigs started to vomit in the first five minutes after the IP challenge, and continuously vomited several times in the next 15–30 min. They were lethargic during this time (Figures 2C,G). Within 15 min of the IP challenge their body temperature dropped significantly in comparison to the control group (Figure 2D). At 60 min post challenge, all pigs presented an intense cutaneous rash (Figure 2G). In one of the pigs the rash dissipated at 120 min post challenge and completely diminished in the next hour. However, another pig still had prominent skin rashes around the neck and head region. At 6 h post IP challenge, skin findings in all pigs returned to baseline, however, they remained lethargic. In contrast to the TS group, none of the pigs in the IP sensitization group had trouble breathing. Therefore, according to our definition of systemic anaphylaxis (which requires the simultaneous presence of symptoms including breathing difficulty, intense skin rash, and impaired mobility), none of the pigs in the IP group were deemed to be experiencing systemic anaphylaxis. In one of the pigs, the body temperature did decline considerably and did not rise back up. This pig (male) died at 8 h post-challenge due to persistent hypothermia despite medical intervention (Figure 2H). The following day, the remaining pigs were normal and healthy without any allergic symptoms.

#### 3.2.3 Second IP challenge

During the second IP challenge, all pigs responded with a reaction, however, the intensity of clinical reactions was less severe as compared to the first IP challenge. The clinical symptoms are discussed below in detail.

*TS sensitized group:* Out of the three, one pig showed cutaneous anaphylaxis 15 min post allergen injection with evidence of slight cyanotic skin discoloration (Figure 2C). The skin erythema remained up to 60 min and completely diminished at 120 min post challenge. In another pig, the skin rashes appeared at 30 min and resolved fully at the 120 min mark. However, in the third pig, the skin rashes appeared later at around 60 min post challenge and resolved fully at the 120 min mark (Figure 2C). All pigs vomited several times and were lethargic. There was a significant drop in body temperature recorded in one of the pigs, which returned to baseline at the 120 min mark (Figure 2D). For the other two pigs, the body temperature remained close to the baseline. The day after the challenge, all pigs were active and normal without any allergic symptoms.

*IP sensitized group:* Two out of the three pigs started vomiting at 15 min post challenge and vomited several times over the next 30 min (Figure 2C). All pigs were lethargic and less active at the 120 min mark. The skin rashes started to appear in two of three pigs at 30–45 min post challenge, however, the rashes faded at the 60 min mark. All pigs returned to normalcy within 120 min of the challenge. A few degrees drop in body temperature was observed; however, the change was not statistically different from the TS group (Figure 2D).

#### 3.2.4 Third Ip challenge

Both groups of sensitized pigs retained hypersensitivity. The details are discussed below.

*TS sensitized group:* One out of the three pigs showed cutaneous anaphylaxis at 15 min post allergen injection (Figure 2C), however, another pig showed just moderate skin rashes in the abdominal area in the first 15 min. The rashes remained up to 30 min and then started to resolve at the 45 min mark. In the third pig, the skin rashes appeared slightly later around 60 min, and persisted till 120 min (Figure 2C). No respiratory distress or systemic anaphylaxis was observed in any of the pigs. The change in body temperature was not considerable (Figure 2D).

*IP sensitized group:* All pigs were active and asymptomatic up to 30 min post challenge. Two out of the three pigs started vomiting at the 60 min mark, and the pigs became lethargic. The skin rashes started appearing on all pigs around 60 min post challenge and resolved at the 120 min mark (Figure 2C). None of the pigs experienced respiratory distress, and no significant drop in body temperature was observed (Figure 2D).

Overall, while the oral PE challenges had no significant impact, the IP PE challenges led to varying degrees of clinical symptoms including systemic anaphylaxis and death in the TS sensitized group, and a death in the IP sensitized group due to hypothermia (but without respiratory distress). The different clinical symptoms after the 1st, 2nd, and 3rd IP challenges are summarized in Figure 2I.

### 3.3 Serological markers

**Figure 3:**
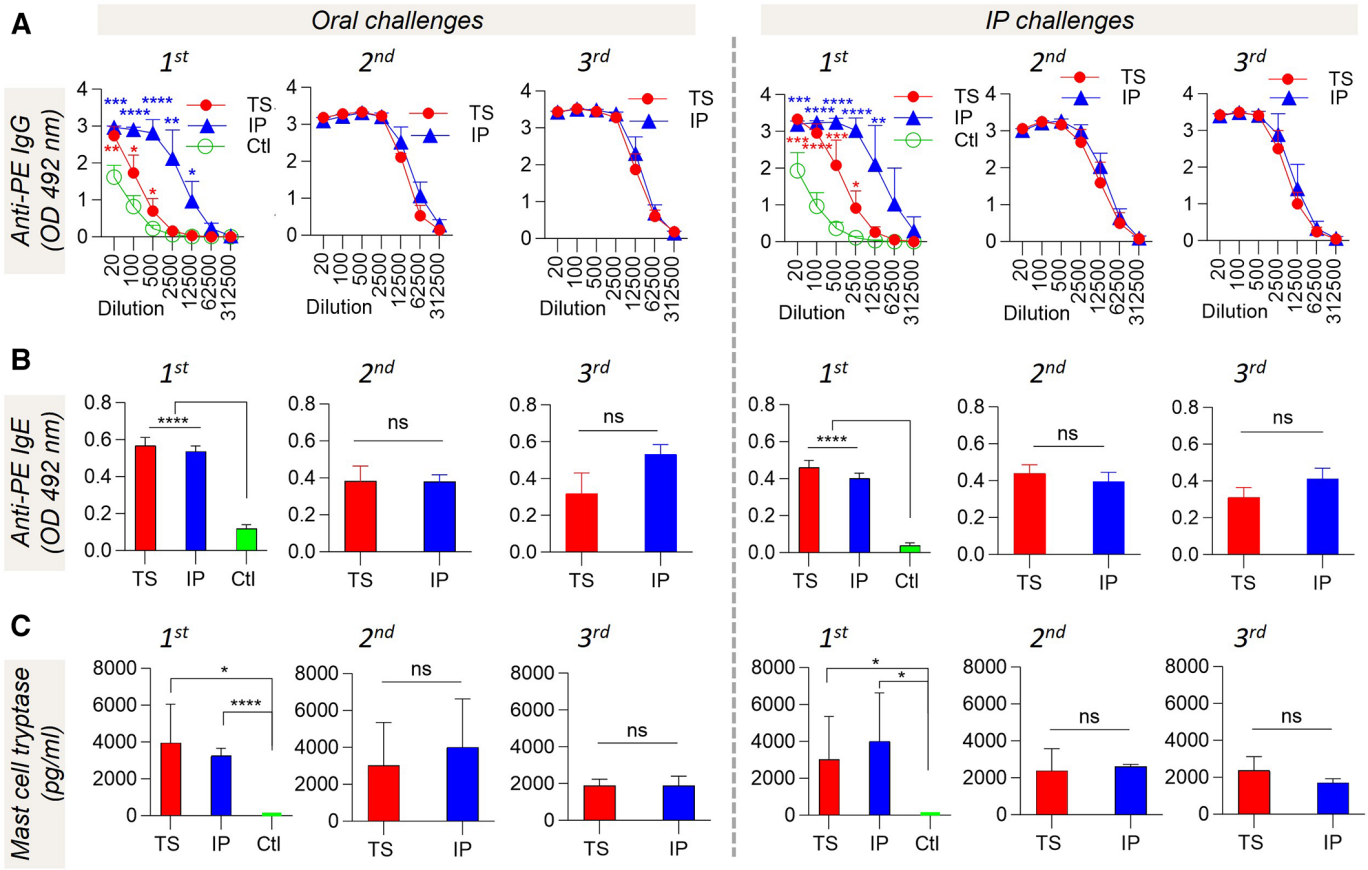
Plasma analysis after different oral and IP challenges. Plasma levels of (**A**) PE-specific IgG, (**B**) PE-specific IgE at 1:20 plasma dilution, and (**C**) mast cell protease at 1:10 plasma dilution. Individual pig plasma was used for analysis. The *t*-test was used to compare sensitized and control pigs. Bars denote Mean ± SD. *p ≤ 0.05, **p ≤ 0.01, ***p ≤ 0.001, ****p ≤ 0.0001, and ns: not significant. *N* = 4 pigs per group for 1st challenge. *N* = 3 pigs per group for 2nd and 3rd challenges. The control (Ctl) group is only presented in the 1st challenge. This is because, during the 1st challenge, the Ctl group experienced an oral challenge, IP challenge, and ISHT. Consequently, it is no longer deemed naïve to PE exposure and is no longer suitable to function as a Ctl group.

PE-specific IgG (Figure 3A) and IgE (Figure 3B) were elevated in both TS and IP groups in comparison to the control group, and this response persisted even after second and third oral and IP challenges. Besides the antibody response, mast cell tryptase was also detected in pig plasma collected after different challenges. Significantly higher mast cell tryptase concentration was found in both TS and IP pigs (Figure 3C). The levels of anti-PE IgE (Figure 3B) and mast cell tryptase (Figure 3C) demonstrated a diminishing trend from the 1st to the 2nd and then to the 3rd challenge with PE.

### 3.4 ISHT response

ISHT against PE was evaluated a week after each IP challenge. Histamine was used as a positive control, and rice protein and saline as the negative controls.

**Figure 4:**
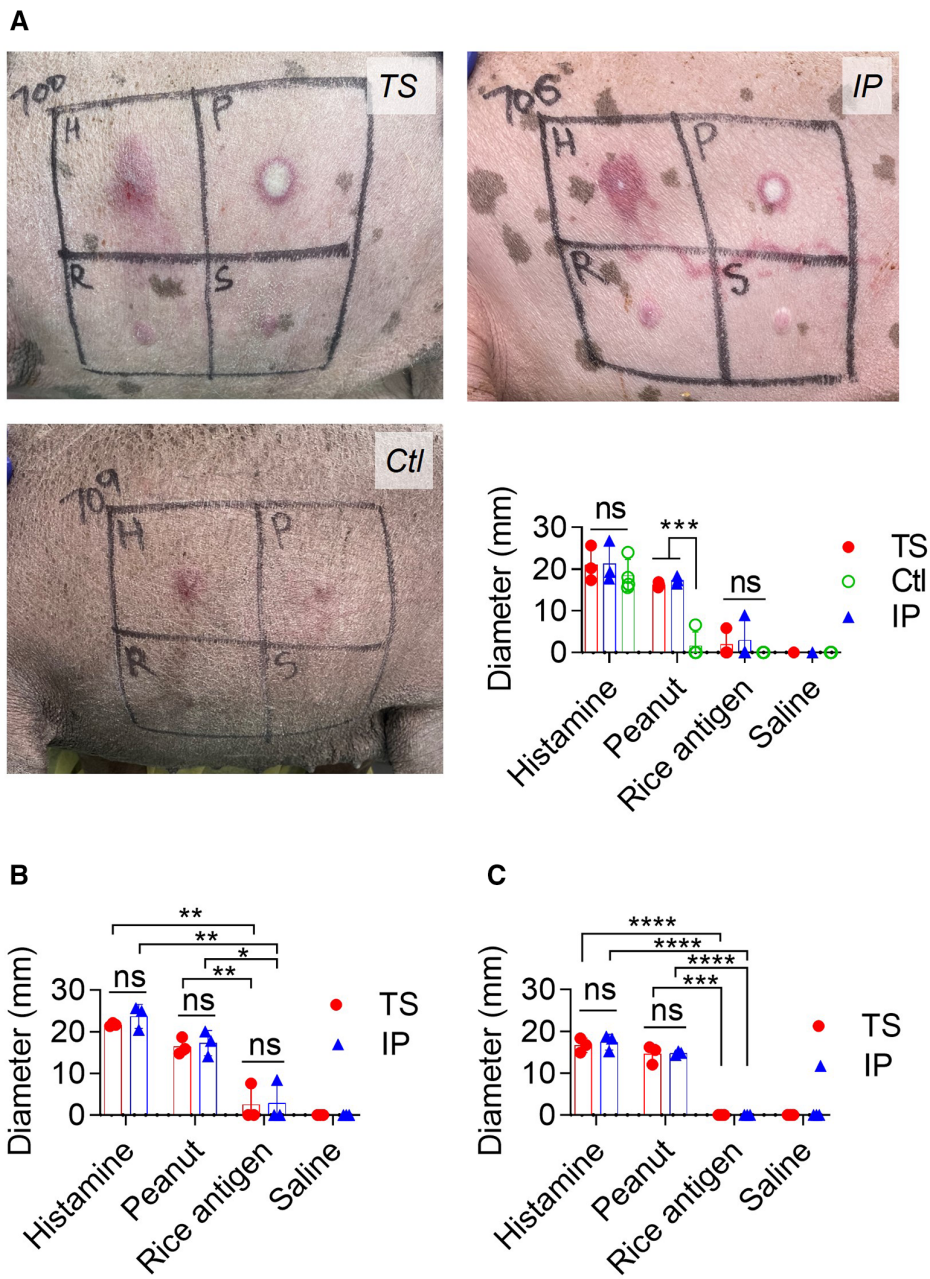
Immediate skin hypersensitivity assessment: (**A**) pictures showing skin hypersensitivity reaction and WFD from 1st skin test. H: histamine, P: Peanut protein extract, R: rice protein and S: saline solution. (**B**) WFD from 2nd skin test. (**C**) WFD from 3rd skin test. The *t*-test was used to compare TS and IP-sensitized pigs. Bars denote Mean ± SD. *p ≤ 0.05, **p ≤ 0.01, ***p ≤ 0.001, ****p ≤ 0.0001, and ns: not significant. Since one pig died in each of TS and IP sensitization groups during the 1st challenge, the TS and IP groups have *N* = 3 pigs each while the Ctl group has 4 pigs. The control (Ctl) group is only presented in the 1st challenge. This is because, during the 1st challenge, the Ctl group experienced an oral challenge, IP challenge, and ISHT. Consequently, it is no longer deemed naïve to PE exposure and is no longer suitable to function as a Ctl group.

*First ISHT:* Figure 4A shows a pictorial and measured WFD response in the TS, IP and Ctl groups after the first ISHT. In the TS group, the WFD from PE, histamine, and rice protein injection was 16 ± 0.6 mm, 21 ± 4 mm, and 2 ± 3 mm, respectively, while for saline it was negligible and assigned a value of zero. The IP-sensitized pigs also showed a positive reaction with a WFD of 21 ± 5 mm, 17 ± 1 mm, and 3 ± 5 mm from a PE, histamine, and rice protein injection, respectively. The reaction spots from PE injection persisted until 72 h post-injection.

*Second ISHT*: Both TS and IP groups of sensitized pigs retained peanut hypersensitivity during their second ISHT (Figure 4B). In TS pigs, the WFD was 16.5 ± 2 mm for PE, 21.7 ± 0.4 mm for histamine, 2.5 ± 4.4 mm for rice protein, and negligible for saline injection. Likewise, IP pigs had a WFD of 17.3 ± 3 mm for PE, 23.7 ± 3 mm for histamine, 2.8 ± 4.9 mm for rice protein, and there was no reaction to saline injection.

*Third ISHT:* Peanut hyperreactivity was observable even in third ISHT (Figure 4C). In TS pigs, the WFD was 14.6 ± 2.2 mm for PE, 16.6 ± 1.7 mm for histamine, and negligible for rice protein and saline injections. Likewise in IP pigs, the WFD was 14.8 ± 0.5 mm for PE, 17.4 ± 1.8 for histamine, and negligible for rice protein and saline injections.

## 4 Discussion

A procedure for establishing a pig model of peanut allergy must be consistent. In other words, the majority of pigs in the treated group should develop sensitization, and their allergy symptoms should manifest to a similar extent. Furthermore, it is preferable that their allergy symptoms should include some of the severe clinical indicators seen in humans, including anaphylaxis.

Helm et al. (8) have previously reported a pig model of peanut allergy in farm neonatal pigs by sensitizing them through the IP route. Prior to establishing the protocol detailed in this study, we initially started (Supplementary Material**)** by following the procedure outlined by Helm et al. However, upon conducting an oral challenge with peanut proteins on the treated pigs, we observed only mild allergy symptoms, and the proportion of responsive individuals was also limited (Supplementary Table S1**)**. To improve consistency, we made numerous modifications to their protocol such as using the oral route and oral + IP route for sensitization besides the IP route alone, and performing an IP challenge with peanut proteins in addition to the oral challenge. The IP route of challenge directly introduces peanut proteins into the systemic compartment, and it should elicit a more heightened allergic reaction (12). Despite these modifications, none of the pigs exhibited severe allergic reactions, and the number of responders remained minimal. It is noteworthy that in some cohorts we observed almost no allergic reaction even after IP challenges. A similar observation was made by Rupa et al., who noted that in their egg allergy model in pigs, out of three litters they attempted, one simply failed to produce sensitized pigs (10).

Following several unsuccessful outcomes using the Helm et al. procedure, our aim in this study was to establish a more reliable pig model of peanut allergy. To achieve this objective, we compared the IP route to the topical skin route for allergen sensitization. We chose the miniature porcine strain Yucatan over the domestic farm strains such as Landrace due to its ease of handling and slower growth rate, allowing the studies to be more conveniently performed for longer durations. The oral route of sensitization was not considered because our studies in Landrace pigs had revealed no significant benefit of using the oral over the IP route for inducing sensitization against peanut (Supplementary Table S1). The skin serves as a unique immunological organ that acts as a protective barrier against microbe entry, prevents water loss, and prevents the entry of various allergens from the environment (13, 14). Recently, mechanically compromised skin has been utilized as an alternative method to induce food sensitization and provoke anaphylactic reaction in rat and mice (15, 16). In humans, excessive tape-stripping has also been implicated in allergy exacerbation rather than achieving a therapeutic effect (17). Hence, we employed the tape-stripping process to mechanically disrupt the skin barrier and tested its ability to induce peanut sensitization in Yucatan mini pigs.

Previously, Helm et al. used five IP injections (three on consecutive days followed by two more weekly injections) (10), while Rupa et al. gave three injections separated by 7 and 14 days (11). We postulated that since the activation of the immune response is not an immediate process, the IP injections should be separated by at least a week. Furthermore, we increased the number of IP injections with the hypothesis that doing so would enhance peanut-specific IgE titers and heighten the severity of allergic hypersensitivity. Accordingly, we tested a protocol of six IP injections each spaced one week apart. To implement the skin-based sensitization, we carefully developed a system of tape-based reservoirs and covers, designed the system to keep the liquid allergen in contact with TS skin for a prolonged period of about 24 h. Similar to Helm et al., we chose CT as an adjuvant for sensitization because it is known for stimulating a type 2 inflammatory response and generating an allergy-inducing immune response when combined with the allergen (18).

To test the result of IP and TS sensitization protocols, we challenged the Yucatan pigs with peanut proteins through two different routes, namely, the oral and the IP route. While the oral route is arguably the most relevant route for food challenge, it failed to induce an allergic reaction in Yucatan pigs despite the presence of systemic peanut-specific IgE antibodies. This could be attributed to various factors such as differences in food metabolism between pigs and humans and an insufficient level of severity generated by our sensitization protocols. In contrast, challenge with peanut proteins through the IP route resulted in severe allergic symptoms, including systemic anaphylaxis in pigs that were sensitized via the TS route. Although the IP route may not be ideal for mimicking the human allergen challenge, it does offer better control of allergen dosage and ensures consistency in the development of food allergy symptoms.

After IP challenges, we observed pronounced peanut hypersensitivity with a 100% incidence in pigs sensitized via either IP or TS protocols. Ara h1, h2, h3, and h6 are implicated as key proteins contributing to peanut allergies in humans (19, 20). Both TS and IP routes of sensitization triggered IgE responses to Ara h1 and Ara h3 proteins. However, only the IP route elicited a significant IgE response against Ara h2 and a weak stimulation of IgE antibodies against Ara h6. Despite lower IgE responses to Ara h2 and Ara h6 in the TS-sensitized group, this group of pigs experienced breathing difficulty and subsequently systemic anaphylaxis. In contrast, intriguingly, the IP-sensitized pigs did not show respiratory distress. To gain a deeper understanding of this phenomenon, further studies concentrating on molecular and cellular analysis of blood and other tissues are necessary.

Mast cells are pivotal mediators in allergic reactions (21). When activated, they undergo degranulation, releasing various compounds that play a role in the development of diverse allergy symptoms. Among these compounds, mast cell tryptase serves as a commonly utilized biomarker for identifying mast cell degranulation (22). Indeed, mast cell tryptase levels were found to be elevated in all three IP challenges for both TS and IP sensitization groups, indicating the involvement of mast cells in the observed allergic reactions.

Peanut hypersensitivity in sensitized pigs was additionally confirmed through ISHT, which mimics the skin prick test used for diagnosing IgE-mediated food allergies in humans (23). Both TS-sensitized and IP-sensitized groups exhibited a significantly high WFD when injected with PE. The irrelevant rice protein did not result in a significant WFD, indicating the specificity of ISHT to peanut proteins.

An inherent limitation of this study is that each group originally comprised four pigs; however, in both the TS and IP sensitization groups, this number reduced to three per group due to the death of one pig in each group after the initial IP challenge. While these group sizes are on the lower side, it is important to note that all four pigs in both TS and IP groups exhibited significant allergy symptoms, underscoring the robustness of the protocols. The common clinical reactions observed in sensitized pigs included vomiting, lethargy, skin rashes, aggressive scratching, and a significant drop in body temperature. In comparison to earlier porcine studies that documented mild and sporadic allergic reactions in sensitized pigs (8, 9), a 100% of the TS and IP sensitized pigs of this study displayed severe clinical symptoms. These findings, especially in TS pigs are novel and, to the best of our knowledge, have not been reported previously.

In a separate study (data not shown), we have successfully replicated the model and are currently employing it to evaluate the desensitization effect using PE-coated microneedles. In this repeated study, once again, we observe more severe allergy symptoms in the TS-sensitized pigs compared to the IP-sensitized group. As a result, we have confidence in the reproducibility of the protocol. We believe that standardizing the TS process and allergen application using the tape reservoirs contributes to the reproducibility of the TS sensitization process.

Allergic reactions remained inducible for up to two months after discontinuing the allergen sensitization protocol. However, there was a gradual decline in both PE-specific IgE levels and the severity of allergy symptoms over time. Therefore, further research is needed to enhance the model, enabling the sustained induction of severe allergic reaction symptoms, including systemic anaphylaxis, even several months to up to a year after discontinuing sensitization. A decline in anti-PE IgE correlated with milder clinical symptoms of allergic reactions and a decrease in mast cell tryptase in plasma, highlighting the significant role of IgE in the observed allergic reactions. Consequently, it can be postulated that in the future, identifying approaches to further enhance anti-PE IgE responses could be advantageous in sustaining severe allergic reactions. One potential strategy for extending the severity of allergic reactions might involve combining the TS and IP sensitization protocols or increasing the doses of PE and CT used during sensitizations.

## Data Availability

The original contributions presented in the study are included in the article/Supplementary Material, further inquiries can be directed to the corresponding author.
