# Supplementary material for: Development of a mini pig model of peanut allergy
Source: Front Allergy. 2024 Feb 12;5:1278801. doi: 10.3389/falgy.2024.1278801 (PMC10894917; doi:10.3389/falgy.2024.1278801)
Supplement: Supplementary file 1 [file Datasheet1.docx]

**Development of a mini pig model of peanut allergy**

Akhilesh Kumar Shakya^a^, Brittany Backus^b^, Lazar D Nesovic^a^, Malini Mallick^a^, Olivia Banister^a^, Carla M. Davis^c^, Sara Anvari^c^, and Harvinder Singh Gill^a,*^

^a^ *Department of Chemical Engineering, Texas Tech University, Lubbock, TX 79409, USA*

*^b^ Department of Animal and Food Sciences, Texas Tech University, Lubbock, TX 79409, USA*

*^c^ Division of Immunology, Allergy and Retrovirology, Baylor College of Medicine, Texas Children’s Hospital, Houston, TX 77030, USA*

**Running title:** Peanut allergy animal model

***Corresponding author:**

Dr. Harvinder Singh Gill

Texas Tech University

Department of Chemical Engineering

E-mail address: harvinder.gill@ttu.edu (H.S. Gill).

**Supplementary Material**

**Methods**

The protocol of Helm et al.[1] was followed to induce peanut allergy in pigs. Briefly, Large White x Landrace newborns were obtained from Texas Tech University Swine Farm. They were sensitized via the IP route or the oral route or combined IP+oral routes by giving doses on days 9, 10, 11, 18 and 25 after birth. For IP route, alum and CT were tested as adjuvants. In some experiments IP sensitization was performed with 0.5 mg peanut extract (PE) + 100 µg CT, while in other experiments 10 mg alum was used instead of CT. For oral sensitization 1 g peanut flour was mixed with 100 µg CT. For the IP+Oral sensitization approach, the pigs received IP (0.5 mg PE + 100 µg CT) + Oral (1g peanut flour + 100 µg CT) doses. Oral sensitization was performed using oral gavage with a feeding tube.

Peanut challenges were performed through oral and IP routes on day 39. For oral route, two doses were tested, 20 g peanut flour and 25 g peanut flour. For IP route the pigs received 0.2 g PE. After the challenge pigs were monitored for clinical symptoms and change in body temperature for 120 minutes. Skin test was performed one week later by injecting 100 µg PE intradermally.

**Results**

Results are summarized in **Table S1**. Six independent experiments were performed.

Experiment 1, was performed as per the protocol of Helm et al.[1]. Pigs received IP injection of 0.5 mg PE + 100 µg CT. As a control we also included an oral group receiving 1 g peanut flour + 100 µg CT. Upon oral challenge the pigs showed no significant allergic symptoms. Mild skin rash was observed in two pigs of IP sensitization.

Due to the lack of significant allergic reactions observed in most pigs during Experiment 1, we conducted Experiment 2, comparing CT with alum as an adjuvant. We hypothesized that alum might elicit a more robust sensitization reaction based on its successful use in developing airway allergy models in mice[2]. Since Experiment 1's IP sensitization had only two mild responders, we introduced an IP group (with alum as adjuvant) and explored an IP+Oral sensitization group (with CT as adjuvant). Despite these variations, post-oral challenge, none of the pigs exhibited noteworthy signs of allergic response.

In Experiment 3, we reverted to using CT as an adjuvant and compared IP (CT as adjuvant) and IP+Oral (CT as adjuvant) routes of sensitization. Following oral challenge, only one pig in the IP+Oral group displayed a mild reaction, while the remaining pigs showed no reactivity.

In Experiments 4, 5, and 6, we made multiple attempts to induce sensitization in pigs. However, the responses were sporadic, with most pigs failing to react significantly to an oral challenge or an IP challenge, albeit the IP challenge seemed to induce slightly stronger allergic reactions.

**Table S1. Summary of clinical symptoms in Large White x Landrace pigs after sensitization and challenge with peanut proteins.**

| **Expt. No. (starting date)** | **Sensitization route** | **Pig strain** | **Challenge route** | **Challenge outcome** |
| --- | --- | --- | --- | --- |
| Expt. 1 (01/2018) | *IP (n=5),  ^$^Oral (n=5),  Ctl (n=2) | Large White x Landrace | **^ϕ^**Oral | - Mild skin rashes were observed in two IP pigs  - All IP group pigs were positive for skin hypersensitivity test  - None of oral pigs responded to the peanut challenge |
| Expt. 2 (03/2018) | **IP (n=3),  ^$$^IP+Oral (n=3),  Ctl (n=2) | Large White x Landrace | **^ϕϕ^**Oral | - No group of sensitized pigs responded against the peanut challenge |
| Expt. 3 (02/2019) | *IP (n=6),  ^$$^IP+Oral (n=6),  Ctl (n=3) | Large White x Landrace | **^ϕ^**Oral | - Mild allergy symptoms like lethargy, swollen eyes, drooling were observed in only one IP+oral sensitized pig  - No allergy symptoms observed in IP group  - Both sensitized groups of pigs showed positive reaction during skin hypersensitivity test |
| Expt. 4 (05/2019) | *IP (n=3),  ^$$^IP+Oral (n=3) | Large White x Landrace | **^ϕ^**Oral then ^§^IP | - Mild allergy symptoms including vomiting, diarrhea, and skin rash were observed in one of the IP+Oral sensitized pigs during oral challenge however none of them responded to the IP challenge  - Both sensitized group of pigs showed positive reaction during skin hypersensitivity test |
| Expt. 5 (08/2019) | *IP (n=6),  Ctl (n=5) | Large White x Landrace | **^ϕ^**Oral then ^§^IP | - Mild allergy symptoms like swollen eyes, lethargy were observed in two IP sensitized pigs during IP challenge however none of the pigs responded to the oral challenge  - Sensitized pigs showed positive reaction during skin hypersensitivity test |
| Expt. 6 (11/2019) | *IP (n=7),  Ctl (n=3) | Large White x Landrace | **^ϕ^**Oral then ^§^IP | - Moderate allergy symptoms including vomiting, diarrhea, and rash were observed in three IP sensitized pigs during IP challenge however none of the pigs responded to the oral challenge  - IP sensitized pigs displayed positive reaction during the skin hypersensitivity test |

**Sensitization routes**

*IP : 0.5 mg peanut protein extract (PE) + 100µg CT

**IP : 0.5 mg PE+10 mg Alum

^$^Oral: 1 g peanut flour + 100 µg CT

^$$^IP+Oral: IP (0.5 mg PE + 100 µg CT) + Oral (1g peanut flour + 100 µg CT)

**Challenge doses**

**^ϕ^**Oral: 20 g peanut flour

**^ϕϕ^** Oral**:** 25 g peanut flour

^§^IP: 0.2 gm PE

**References**

1. Helm, R.M., Faruta G.T., Stanley J.S., Ye J., Cockrell G., Connaughton C., et al., *A neonatal swine model for peanut allergy.* J Allergy Clin Immunol, 2002. **109**(1): p. 136-42.

2. Eisenbarth, S.C., *Use and limitations of alum-based models of allergy.* Clin Exp Allergy, 2008. **38**(10): p. 1572-5.
