# Supplementary figures and images for: Development of a mini pig model of peanut allergy
Source: Front Allergy. 2024 Feb 12;5:1278801. doi: 10.3389/falgy.2024.1278801 (PMC10894917; doi:10.3389/falgy.2024.1278801)

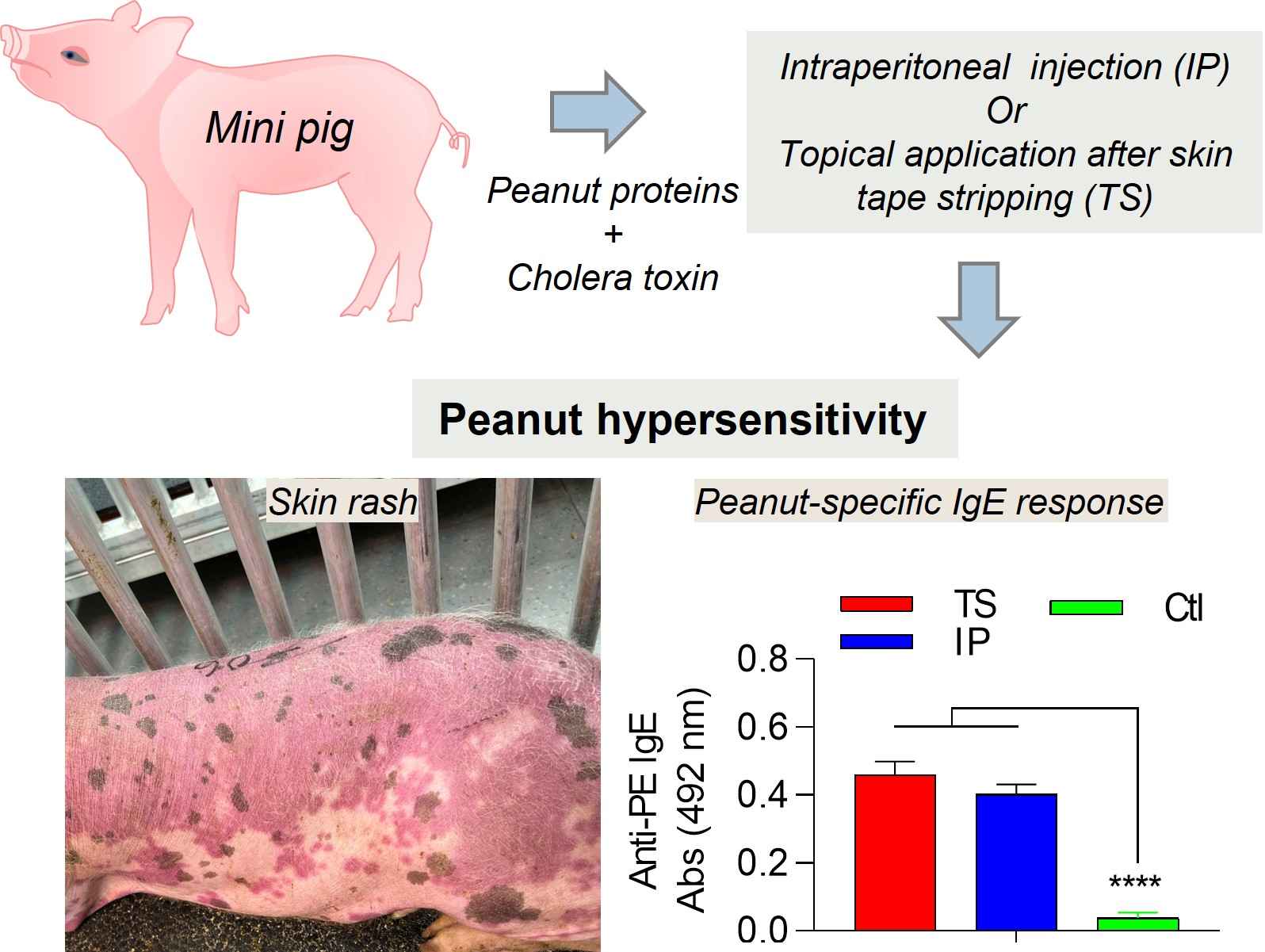

Supplement: Supplementary file 2 [file Image1.jpeg]
